# Patients' Knowledge and Attitude toward the Disposal of Medications

**DOI:** 10.1155/2017/8516741

**Published:** 2017-10-10

**Authors:** Aeshah AlAzmi, Hani AlHamdan, Rayf Abualezz, Faiz Bahadig, Noha Abonofal, Mohamed Osman

**Affiliations:** ^1^Ministry of National Guard Health Affairs (MNGHA), King Abdulaziz Medical City (KAMC 6255), Pharmaceutical Care Services Department, P.O. Box 9515, Jeddah 21423, Saudi Arabia; ^2^Ministry of National Guard Health Affairs (MNGHA), Prince Mohamed Bin Abdulaziz Hospital, Pharmaceutical Care Services Department, P.O. Box 40740, Madinah 41511, Saudi Arabia

## Abstract

**Background:**

Safe disposal of medications is of high concern as malpractice may lead to harmful consequences such as undesirable effects, prescription drug abuse, overstocking, self-medication, accidental overdose, and even death. There is a lack of uniform and nationwide guidance on how patients should safely dispose their leftover medications. This study aims to assess patients' knowledge and attitude regarding the disposal of medications.

**Method:**

This research is a cross-sectional study. A self-administered questionnaire was used to collect data from various outpatient pharmaceutical services in King Abdulaziz Medical City (KAMC), Jeddah.

**Results:**

The study revealed that 73% of the respondents throw the medications in the trash, 14% return the medications to a pharmacy, 5% never dispose them, and 3% donate the medications to a friend or charity centers. More than 80% of the respondents never received any information or advice from healthcare providers about safe and proper disposal of medications.

**Conclusion:**

Our findings suggest that there is an immediate requirement for the establishment of collaborative and uniform guidelines for the safe disposal of leftover medications. A policy for drug donation needs to be included in routine patient education as well as educational and collective programs for the public.

## 1. Introduction

There is a lack of knowledge on how to dispose expired, unwanted, and unused medications among our participant population. Some international and national studies showed that most patients stored their medications improperly at home that may lead to undesirable effects or unintentional risks like improper self-medication, accidental overdose, and prescription drug abuse. These patients keep medications because they do not want to waste them, they do not know how to read and check the expiry date, or they do not know a proper and safe way to dispose them [1–5].

Expired medications, as reported in many studies, were either flushed down the sink, drain, or toilet or thrown into the trash or garbage. According to the study by Abou-Auda (2003), people of gulf countries tend to store or throw away most of the unused or expired medications [1]. Similar findings were recently published in Riyadh by Al-Shareef et al. [6] Abahussain and Ball conducted a questionnaire-based study in Kuwait (2007) and found that 77% of the respondents disposed their medications in the garbage, 12% returned the medications to government health centers, and almost 50% of the returned medications had expired [7]. From Qatar, Kheir and colleagues (2011) conducted an exploratory study on medications in households and found that the majority of their study population shared prescription medications and disposed unwanted ones in the garbage [8].

There are three major concerns to this issue. First, if drugs are carelessly stored or disposed, there is a likelihood of another person obtaining and consuming them leading to serious consequences and health threats. The American Association of Poison Control Centers' (AAPCC) National Poison Data System (NPDS) (2010) reported that overdose and poisoning deaths accounted for more than 30 percent of accidental ingestion of prescription or over-the-counter medications that led to child fatality [9].

Paulozzi and colleagues found that almost 831,295 visits to an Emergency Department were due to unintentional poisoning [10]. In a series of tragedies, exposure to an opioid medicine discarded in the trash or carelessly stored was ingested by a child that resulted in poisoning, and in some extreme cases, fatalities were also reported [11, 12]. Storing medication in child resistant containers cannot completely prevent a child from accessing the medicine. This was the result from a study that examined cases of accidental exposure of a child to medicines, and it was found that nearly 50% of grandparents' medicines were stored in child resistant containers [13]. Second, some of the medications, if flushed down a drain or toilet, may not be destroyed adequately by the sewer system and may cause harm to plants and animals and can enter the water supply system meant for consumption [14]. In a study conducted in United States (US), researchers found a considerable level of chemical compounds, commonly found in medications, in 80% of the sampled streams [15]. Several studies found the same in fresh drinking water [16–18]. Similar results were obtained from a study conducted in Europe. Considerable levels of pharmaceutical elements were found in the environment including groundwater, sewage, and drinking water [19–29]. Although low concentration of these medications is negligible, the existence of multiple compounds in water and the environment can cause serious health threats in the long run [30, 31]. Third, medication wastage considered a universal problem and has a significant impact on the cost of healthcare. Under Saudi Arabian law, all Saudi citizens have the right to receive medicines from all healthcare facilities free of charge [32]. Non-Saudi patients may obtain basic medicines free of cost from all healthcare facilities [33]. Some patients choose to give away their unused/unwanted medication by donating it to family or friends that can be a harmful risk instead of a helpful purpose [34].

The World Health Organization (WHO) in 1996, 1999, and 2010 published evidence-based guidelines to control the practice of drug donations and empower recipients. Many reasons stated in the guidelines support the unnecessary/unsuitable donation of unused/unwanted medications that may lead to its inappropriate use. Since the quality of donated drugs cannot be guaranteed, it is clearly stated in the guidelines that “No drugs should be donated that have been issued to patients and then returned to a pharmacy or elsewhere, or were given to health professionals as free samples” [34]. Ette looked at medication donation cases and found that developing a one-year shelf-life guideline was important to control the factors that influence drug benefit (storage and sorting) [35, 36]. Another method of disposal is to return expired, unwanted, or unused medication to a local pharmacy and clinic or to a healthcare provider for safe disposal.

In fact, few pharmacies willingly take back such medications for proper disposal. The pharmaceutical care services department at KAMC, Jeddah, has a policy that allows the receipt and disposal of returned medications from patients, but so far, patients were seldom aware of the existence of this service. Patients usually receive instructions on how to use and store medications but rarely do they get proper information on the safe and appropriate ways to dispose such medications, unless they asked the healthcare providers. Seehusen and Edwards (2006) identified a similar problem. They surveyed 301 patients in an outpatient pharmacy in the United States and found that less than 20% patients were advised about the proper disposal of such medications [37].

In the Kingdom of Saudi Arabia, the knowledge of the problem of various drug disposal methods is limited and somewhat unknown. Safe disposal of expired, unwanted, or unused medications is of high concern, as malpractice may lead to harmful consequences. Thus, we conclude that this problem must be prevented. We conducted this study to explore the knowledge and practice toward disposal of medication among patients at various outpatient services in King Abdulaziz Medical City (KAMC), Jeddah.

## 2. Methods

This research was a cross-sectional study and used a self-administered questionnaire to gather data over a period of four weeks. Data were collected from four main outpatient pharmacy services including the Ambulatory Care Center (ACC), Oncology department (ONC), Cardiac Center (CC), and Emergency Department (ED) in KAMC, Jeddah. The survey questions were designed based on international guidelines and frequently asked questions about proper and safe disposal of expired, unwanted, or unused medication [37–51]. The questionnaire was developed in two languages, English and Arabic, and participants could choose either version based on their preference. Based on the results of a pilot study conducted on fifty randomly selected participants, questions were modified to ensure clarity, simplicity, and understanding. The average time taken to complete and return the questionnaire was around 10 minutes. The final modified questionnaire contained two parts. Part I included basic demographic information such as sex, age, and educational level; and part II consisted of eight questions to assess participants' knowledge and attitude toward medication disposal. These questions are “Do you or anyone in your household have medications that are no longer needed?” “When do you think you need to dispose leftover medications?” “How did you or your family handle/dispose unused, expired, or unwanted medications?” “If you store or keep them, what are your reasons for keeping/storing the unused drugs and not disposing them?” “Do you have any concerns regarding your current method of disposal of expired or unwanted medications?” “In your opinion, what is the proper way to manage and dispose unused/unwanted and expired medications?” “Have you ever received any information about the proper way to dispose unused, unwanted, or expired medications?” “In your opinion, what is the best way to educate the public?” The questionnaire was distributed by study surveyors who were 6th grade pharmacy interns and pharmacy residents, under the supervision of one of the study authors. The surveyors were assigned in the different outpatient waiting areas described previously.

*Participant Recruitments.* Participants were randomly selected without any specific methods of recruitment. Any individual entering any of the selected outpatient pharmacy waiting areas during working hours (08:00–17:00) on weekdays was invited to participate in the study. The participants are comprised of patients, family members, and working staff, aged 16 years and older (in our institution adult services are provided to people older than 15 years), and who were able to communicate and understand questions. Incomplete questionnaires and people who did not meet the inclusion criteria were excluded. Study surveyors approached the participant, introduced himself/herself, and informed them about the purpose of the study. They then explained the meaning of medications as prescription drugs, over-the-counter drugs, supplements, vitamins, and herbal products and obtained verbal consent of the person to participate in the study. Once the surveyors distributed the questionnaires, they were not allowed to intervene or answer any of the questions on behalf of the participants, and they collected the questionnaires immediately after the test.

KAMC, Jeddah, is a 751-bed, tertiary care hospital and provides medical services for the Saudi Arabian population in the western region. The pharmaceutical care services department at KAMC, Jeddah, provides pharmacy outpatient services in four different areas (ACC, ONC, CC, and ED) and receives at least 2500 patients on a monthly basis. Descriptive statistics were used to categorize patients' knowledge and attitudes while quantitative variables were summarized with proportions and percentages using the SPSS software.

## 3. Ethical Consideration

This project received approval from the institutional review board of King Abdullah International Medical Research Center Protocol # RJ13/001/J in an approval document memo, dated 2014.

## 4. Result

A total of 1171 participants completed the questionnaire (ACC = 902, ONC = 187, CC = 55, and ED = 27). We did not calculate response rate as the total number of patients who entered the study areas during data collection was not obtained.  Table 1 provides details on the participants' demographic data including age and educational level. The response of female participants was slightly higher than male participants (604/1171, 52% and 567/1171, 48%, resp.). Of the invited participants, 30% were aged 26 to 35 years and 23% were aged between 36 and 45 years. About 36% of the participants held bachelor degrees and nearly 35% had an intermediate and secondary school qualification.

### 4.1. Participants' Disposal Practice

There were six main disposal methods practiced by the respondents. Across all study areas, throwing leftover medications in the trash or garbage was the most commonly practiced method, as reported by the majority the respondents (72.8% 853/1171). This was followed by 13.6% (159/1171) participants who reported returning unused and unwanted medications to a pharmacy, 5.3% (62/1171) participants reported never disposing their medications and storing them at home, 4.6% (54/1171) flushed the medications down the sink or toilet, 2.6% (30/1171) shared or gave away their medications to other people or to a charity, and 1.1% (13/1171) burned the leftover medications whether they were expired, unwanted, or unused.

### 4.2. Practice of Disposing Expired, Unwanted, or Unused Medications among Participants according to Study Areas

#### 4.2.1. Ambulatory Care Center (ACC)

A total of 902 participants completed the questionnaire from the ACC pharmacy waiting area. Nearly 75% (673/902) reported throwing away leftover medications in the garbage whether they were expired, unwanted, or unused; 4% (40/902) reported flushing them down the sink or toilet; 12% (109/902) reported returning them to a pharmacy; 3% (22/902) reported sharing them with friend or giving them away to a charity center; 5% (48/902) reported storing the unused and unwanted medications; and 1% (10/902) reported burning them.  Table 2 provides details of the practice methods of disposing expired, unwanted, or unused medications by participants of the ACC area.

#### 4.2.2. Oncology Department (ONC)

A total of 187 participants completed the questionnaire from the ONC pharmacy waiting area. Nearly 68% (127/187) reported throwing away leftover medications in the garbage whether expired, unwanted, or unused; 4% (8/187) reported flushing them down the sink or toilet; 22% (40/187) reported returning them to a pharmacy; 1% (2/187) reported sharing them with friends or giving them away to a charity center; and 5% (10/187) reported storing the unused and unwanted medications at home.  Table 3 provides details of the practice methods of disposing expired, unwanted, or unused medications by the participants in the ONC area.

#### 4.2.3. Cardiac Center (CC)

A total of 55 participants completed the questionnaire from the CC pharmacy waiting area. Nearly 60% (33/55) reported throwing leftover medications in the garbage whether expired, unwanted, or unused; 7% (4/55) reported flushing them down the sink or toilet; 13% (7/55) reported returning them to a pharmacy; 9% (5/55) reported sharing them with friends or giving them away to a charity center; 7% (4/55) reported storing unused and unwanted medications at home, while 4% (2/55) reported burning them.  Table 4 provides details of the practice methods of disposing expired, unwanted, or unused medications by the participants in the CC area.

#### 4.2.4. Emergency Department (ED)

A total of 27 participants completed the questionnaire from the ED pharmacy waiting area. About 74% (20/27) reported throwing leftover medications in the garbage whether expired, unwanted, or unused; 8% (2/27) reported flushing them down the sink or toilet; 11% (3/27) reported returning them to a pharmacy; 4% (1/27) reported sharing them with friends or giving them away to a charity center; and 4% (1/27) reported burning them.  Table 5 provides details of the practice methods of disposing expired, unwanted, or unused medications by the participants of the ED area.

### 4.3. Participants' Opinion about the Proper Way of Disposing Leftover Medications

More than 40% (480/1171) respondents believed that it was acceptable to store medications at home for future use. When asked what the best way to dispose such medications was, more than 70% respondents said that the best way to dispose leftover medications was by returning them to a healthcare facility or healthcare providers. Study participants reported their need to have secure containers to collect left over medications from private pharmacies, governmental hospitals, and primary care clinics. Due to the distance of the Ministry of National Guard Health Affairs (MNGHA) from the center of Jeddah, participants reported facing a difficulty in transportation to the hospital just for returning the leftover medications. This is founded to be the main barrier for not returning medications to the KAMC, Jeddah pharmacy. In addition to that, local government pharmacies, clinics, and hospitals were not comfortable accepting returned medications dispensed from other facilities and that they were unaware of the storage conditions and lacked a supporting disposal policy. Half of the respondents (50%) thought the right method of disposing medications was to give them to charity institutions like ZmZm association (a charity organization that operates under the umbrella of social affairs) or give them to poor people or non-Saudi citizens who were not eligible for government treatment or could not afford treatment.

#### 4.3.1. Education Concerning Medication Disposal

More than 80% (967/1171) respondents across all study areas reported that they never received any information or advice about safe and proper disposal of medication by their healthcare providers.  Table 6 provides details on education concerning the appropriate disposal of medications.

#### 4.3.2. Leftover Medications That Are Not Disposed by Study Participants

Participants reported that 1864 drugs belonging to nine different medication categories were stored at home. It is of special concern that many participants reported having opioids, chemotherapeutic, anticoagulants, and blood thinner drugs at home.

**Figure 1:**
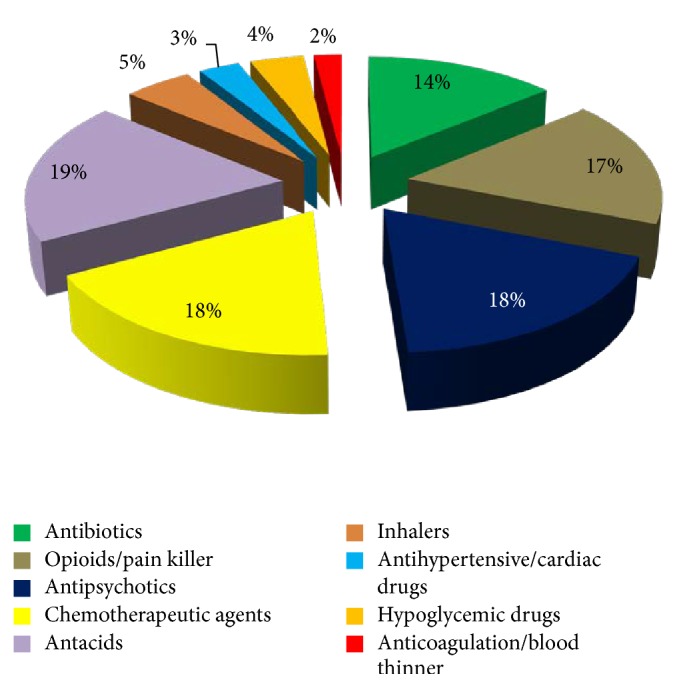
Medication categories (%) of drugs from the Ambulatory Care Center (ACC).

**Figure 2:**
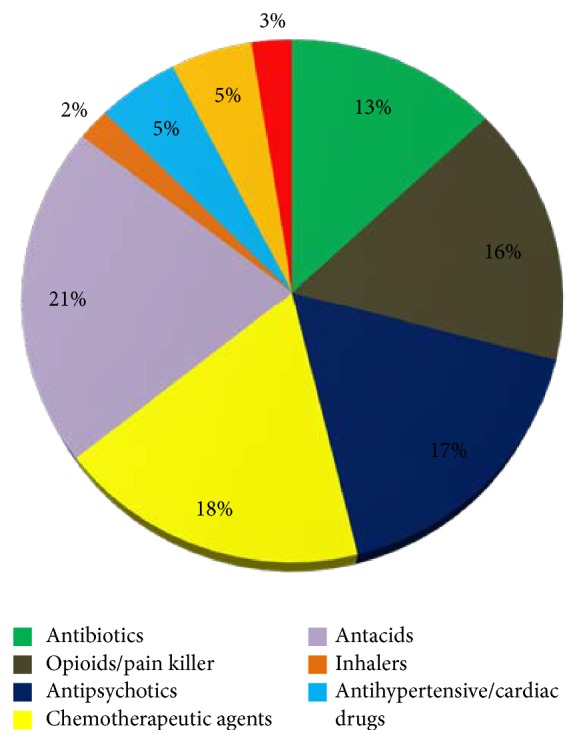
Medication categories (%) of drugs from the Oncology Department (ONC).

**Figure 3:**
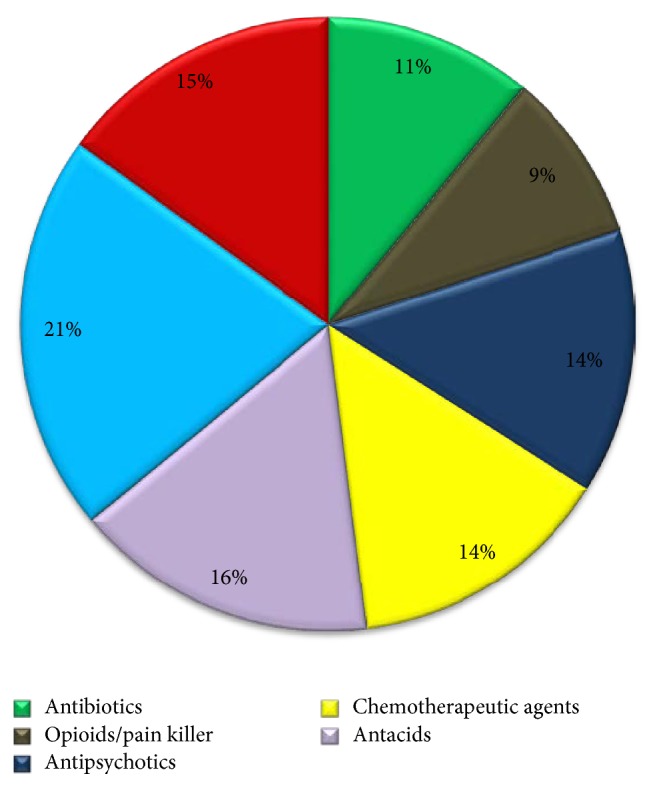
Medication categories (%) of drugs from the Cardiac Center (CC).

**Figure 4:**
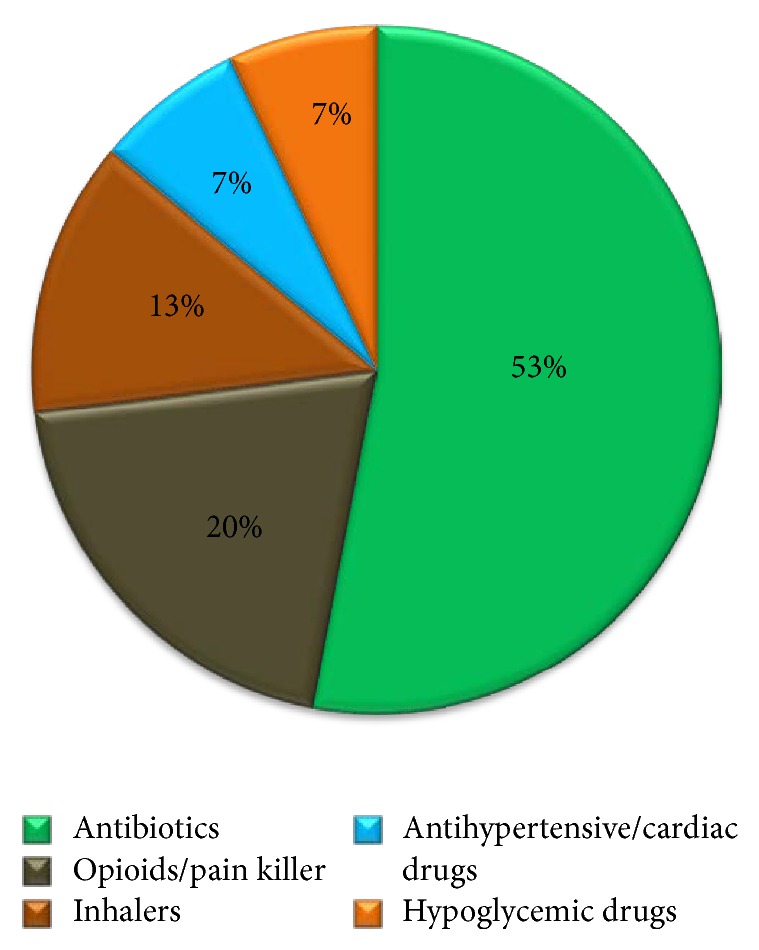
Medication categories (%) of drugs from the Emergency Department (ED).

 Figures 1–4 provide details about medications kept at home by respondents in each study area.

## 5. Discussion

In Saudi Arabia, there is neither a uniform or standard system that accepts and collects expired, unwanted, or unused medications, nor established recycling systems for medication disposal.

Our study showed that nearly 73% of the participants discarded leftover, expired, unwanted, or unused medications by throwing them in the garbage or trash while 50% believed that giving leftover medications away to family, friends, or charity centers is the best method of disposal. Similar findings were reported in many studies. The number of respondents in Riyadh and Kuwait who disposed their medication in the trash was found to be 79% and 77%, respectively [6, 7]. In the United Kingdom (UK), more than 60% of respondents discarded unwanted medications in the trash, and 22% returned them to a pharmacy [52].

Our study showed that the second commonly practiced method of discarding leftover medications was returning them to a pharmacy (14%). Some respondents reported that they store medications in the fridge or closet and never dispose them (5.2%). Flushing medications down the sink or toilet was found to be the third common disposal practice (4.6%). However, different findings were reported in other studies. In Riyadh, UK, USA, and Kuwait flushing medications down the sink or toilet was found to be the second most common method of disposal as reported by 7%, 11%, 26%, and 11% of the respondents, respectively [6, 7, 53, 54]. Medication residues are a source of purified wastewater contamination since sewage water sometimes leaks into underground water, and this water is usually used by Jeddah residents for agricultural and irrigation purposes. This is of special concern, especially in Jeddah, since it is Saudi Arabia's second largest city and does not have a fully developed sewer and wastewater system.

Many respondents in our survey thought that throwing medications in the garbage was more environment friendly than flushing them down the sink or toilet. A study looked at the environmental impact of different medication disposal methods including incineration, flushing, and throwing it in the trash. The authors found that drugs entering the environment through flushing cause air pollution [55]. When we asked the participants about the type of leftover medications kept at home, 15% of the CC respondents said they have anticoagulation drugs and blood thinners at home, and throwing them in the garbage was the most common practice (60%).

From the ONC department, 18% respondents had oral chemotherapeutic drugs, 16% had opioids/pain killers, and 3% had anticoagulation drugs or blood thinners. Throwing them in the garbage was the most common practice of disposing these drugs (68%) while 5% said they never dispose their medication, and 3% said they share them with others.

Opioids, anticoagulants/blood thinners, and chemotherapeutic drugs are hazardous agents. The Environmental Protection Agency (EPA) defines hazardous waste as waste with properties that make it dangerous or have the potential to harm human health or the environment [56]. Although there are limited number of studies that address the side effects of oral chemotherapy, exposure to oral chemotherapeutic agents can happen at any stage that include and are not limited to handling, storing, unpacking, and disposing, and so forth [56, 57]. A study published in 2011 looked at this problem and summarized the responsibilities of patients and their caregivers in carefully handling and discarding oral chemotherapy drugs [58].

In general, more than 80% of participants across all study areas said they never received information for the proper and safe disposal of medications. Thus, healthcare providers should be enrolled in educational programs for proper and safe disposal of medication and provide this information to patients when they visit. Pharmacists have a major role in providing proper information concerning safe disposal of medications. A policy for accepting medication returned from patients exists at the pharmaceutical services department, Ministry of National Guard Health Affairs, Jeddah. However, they lack information related to the safe disposal of specific drugs. Our study annotates the awareness of our population, their opinions, and their current disposal practices. Majority of the respondents agreed that an improper disposal may harm children, animals, the environment, and the planet; and they requested a public awareness program for safe and proper disposal of expired, unwanted, or unused medications. Public education is the key to the current problem. If pharmacists provide information on disposal during medication counseling, the patient will develop positive disposal practices. Furthermore, providing disposal information with each dispensed medication label can play an important role in resolving this issue. Another suggested method is to organize collaborative, nationwide, and awareness campaigns utilizing social media. The same suggestion was provided by a recently published national study [6]. All these findings highlight the urgent need to establish a take back program, particularly in Jeddah and in Saudi Arabia in general. In USA, they have a well-established national program for safe disposal of expired prescription drugs through the Drug Enforcement Administrations' (DEA) National Prescription Drug Take Back Initiative. They organize Drug Take Back events (a year-round activity) since 2010, to promote proper drug disposal. Other involved agencies that provide disposal guidelines include White House Office of National Drug Control Policy with inputs from the Food and Drug Administration (FDA) [45], Environmental Protection Agency (EPA) [46], Centers for Medicare and Medicaid Services (CMS) [47], Occupational Safety and Health Administration (OSHA) [48], Food and Drug Administration (FDA) [49–51], and Pharmacy Return and Waste Companies/Reverse Distributors [51]. These international guidelines are available as guidance and provide recommendations on proper and safe disposal of expired, unwanted, or unused medications. Having a national policy for disposal will be a major influence on people's disposal attitude and practices. A take back program will provide timely disposal methods and may lessen likelihood of medication waste going through the sewage system, and hence environmental pollution may be prevented. A good number of respondents in our study believe in donating drugs to charity centers and providing affordable medication to the poor. Despite the good intention, health may be harmed. The establishment of a national policy to control current patients' practice of donating unwanted and unused drugs is of high priority. It might be wise to concentrate on the take back initiative program first and then spread public awareness for the consequences of the current disposal methods on personal health and on the environment as well. Collaboration between the Ministry of Health (MOH) (as its services comprise 60% of the total health services in Saudi Arabia), other government agencies, and private sector agencies are urgently needed to develop a nationwide policy allowing pharmacies to accept returned medication from the public and serve as a guide for proper medication disposal in Saudi Arabia. The Saudi FDA needs to be involved to oversee and monitor the medication disposal program.

Our study adds to what is already known worldwide but we believe that, to the best of our knowledge, our study is among the first few local studies that address and enlighten patients' disposal practices and attitudes in different pharmacy services within the MNGHA.

*Limitations*. There were some limitations to this study. Our data represents a single institution over a defined period, and this study used a self-administered questionnaire that is subject to recall bias of disposal practices. In our study, we aimed to highlight the disposal practices of patients. However, we did not quantify or identify the formulation of disposed medications or asses the waste cost. We only reported the disposal practices of persons visiting the MNGHA KAMC, Jeddah, while there are many other users of MNGHA primary clinics, other government clinics, and private health systems that may follow unsafe disposal practices. However, the present results provide general ideas and a starting point for future well-designed studies highlighting the extent of medication disposal problems, investigating environment effects, quantifying disposed medication formulations, identifying potential risk factors of drug donation, and clinical outcomes of donated drugs.

## 6. Conclusion

Different drug disposal techniques were identified in our study. Our pharmacy department has a policy for returned medication from patients but lacks specific policies for proper and safe disposal of expired, unwanted, and unused medication. Nationwide, we lack uniform guidelines. Almost little to no information was provided to public on the safe disposal of medications. Creation of uniform guidelines for the safe disposal of medications and guidelines to control drug donation is urgently needed and is of high priority. Safe disposal instructions should be provided by all healthcare providers in routine patient education.

## Figures and Tables

**Table 1:** Demographic data of participants from the different pharmacy outpatient study areas.

Demographic data of participants	Study area									
	ACC *n* = 902		ONC *n* = 187		CC *n* = 55		ED *n* = 27		Total	
									*N* = 1171	
Gender	M = 401 (44%)	**F** = 501 **(56%)**	M = 91 (49%)	**F** = 96 **(51%)**	**M** = 49 **(89%)**	F = 6 (11%)	**M** = 26 **(96%)**	F = 1 (4%)	M = 567 (48%)	**F** = 604 **(52%)**
Age (years)										
16–25	77	111	12	10	3	2	1	0	93	123
26–35	100	157	37	30	15	4	10	0	**162**	**191**
36–45	107	111	20	18	10	0	7	1	144	130
46–55	55	77	11	20	8	0	3	0	77	97
56–75	49	38	10	15	11	0	3	0	73	53
≥76	13	7	1	3	2	0	2	0	18	10
Education										
Not stated	6	5	3	14	0	0	1	0	10	19
Never went to school	13	54	0	1	2	0	0	0	15	55
Primary school	30	59	3	14	5	0	1	1	39	74
Intermediate/ Secondary school	152	150	39	29	21	3	12	0	224	182
Diploma	22	18	9	3	3	0	1	0	34	21
Bachelor degree	133	203	29	28	14	4	8	0	**184**	**235**
Postgraduate study	31	25	8	8	1	1	3	0	40	34

M = Male, F = Female, ACC =Ambulatory Care Center, ONC = Oncology, CC = Cardiac Center, ED = Emergency Department.

**Table 2:** Methods of disposal of medications by respondents of the Ambulatory Care Center (ACC).

ACC (*N* = 902)			
Disposal method	Total *n*/*N* (%)	All medications (%)	Expired only (%)
Thrown away in the trash/garbage	673/902 (75%)	419/673 (62%)	254/673 (38%)
Flushed down the sink/toilet	40/902 (4%)	29/40 (73%)	11/40 (27%)
Return to a pharmacy/clinic	109/902 (12%)	92/109 (84%)	17/109 (16%)
Given away to friend/charity	22/902 (3%)	15/22 (68%)	5/22 (23%)
Stored/never disposed	48/902 (5%)	48/48 (100%)	—
Burned	10/902 (1%)	7/10 (70%)	3/10 (30%)

**Table 3:** Methods of disposal of medications by respondents of the oncology department (ONC).

ONC (*N* = 187)			
Disposal method	Total *n*/*N* (%)	All medications (%)	Expired only (%)
Thrown away in the trash/ garbage	127/187 (68%)	94/127 (74%)	33/127 (26%)
Flushed down the sink/toilet	8/187 (4%)	8/8 (100%)	—
Returned to a pharmacy/clinic	40/187 (22%)	33/40 (83%)	7/40 (17%)
Given away to friends/charity	2/187 (1%)	2/2 (100%)	—
Stored/never disposed	10/187 (5%)	10/10 (100%)	—
Burned	—	—	—

**Table 4:** Methods of disposal of medications by respondents of the Cardiac Center (CC).

CC (*N* = 55)			
Disposal method	Total *n*/*N* (%)	All medications (%)	Expired only (%)
Thrown away in the trash/garbage	33/55 (60%)	23/33 (70%)	10/33 (30%)
Flushed down the sink/toilet	4/55 (7%)	4/4 (100%)	—
Returned to a pharmacy/clinic	7/55 (13%)	5/7 (71%)	2/7 (29%)
Given away to friends/charity	5/55 (9%)	5/5 (100%)	—
Stored/never disposed	4/55 (7%)	4/4 (100%)	—
Burned	2/55 (4%)	2/2 (100%)	—

**Table 5:** Methods of disposal of medications by respondents of the Emergency Department (ED).

ED (*N* = 27)			
Disposal method	Total *n*/*N* (%)	All medications (%)	Expired only (%)
Thrown away in trash/garbage	20/27 (74%)	17/20 (85%)	3/20 (15%)
Flushed down the sink/toilet	2/27 (8%)	1/2 (50%)	1/2 (50%)
Returned to a pharmacy/clinic	3/27 (11%)	3/3 (100%)	—
Given away to a friend/charity	1/27 (4%)	1/1 (100%)	—
Stored/never disposed	—	—	—
Burned	1/27 (4%)	1/1 (100%)	—

**Table 6:** Provision of education on safe disposal of medications by healthcare providers.

Study area (*N*)	Percentage of people who received education on medication disposal		
	Yes	Not sure	Never
ACC (902)	112/902 (12%)	37/902 (4%)	753/902 (84%)
ONC (187)	26/187 (14%)	9/187 (5%)	152/187 (81%)
CC (55)	12/55 (22%)	2/55 (4%)	41/55 (74%)
ED (27)	5/27 (18%)	1/27 (4%)	21/27 (78%)
Total (1171)	155/1171 (13%)	49/1171 (4%)	967/1171 (83%)
